# Docosahexaenoic Acid Modulates Paracellular Absorption of Testosterone and Claudin-1 Expression in a Tissue-Engineered Skin Model

**DOI:** 10.3390/ijms222313091

**Published:** 2021-12-03

**Authors:** Andréa Tremblay, Mélissa Simard, Sophie Morin, Roxane Pouliot

**Affiliations:** 1 Centre de Recherche en Organogénèse Expérimentale de l’Université Laval/LOEX, Québec, QC G1J 1Z4, Canada; andrea.tremblay.4@ulaval.ca (A.T.); melissa.simard.6@ulaval.ca (M.S.); sophie.morin.7@ulaval.ca (S.M.); 2 Axe Médecine Régénératrice, Centre de Recherche du CHU de Québec-Université Laval, Québec, QC G1J 1Z4, Canada; 3 Faculté de Pharmacie de l’Université Laval, Québec, QC G1V 0A6, Canada

**Keywords:** skin substitutes, skin barrier function, tight junctions, polyunsaturated fatty acids, docosahexaenoic acid, tissue engineering, lipidomics

## Abstract

Healthy skin moLEdels produced by tissue-engineering often present a suboptimal skin barrier function as compared with normal human skin. Moreover, skin substitutes reconstructed according to the self-assembly method were found to be deficient in polyunsaturated fatty acids (PUFAs). Therefore, in this study, we investigated the effects of a supplementation of the culture media with docosahexaenoic acid (DHA) on the barrier function of skin substitutes. To this end, 10 μM DHA-supplemented skin substitutes were produced (*n* = 3), analyzed, and compared with controls (substitutes without supplementation). A Franz cell diffusion system, followed by ultra-performance liquid chromatography, was used to perform a skin permeability to testosterone assay. We then used gas chromatography to quantify the PUFAs found in the epidermal phospholipid fraction of the skin substitutes, which showed successful DHA incorporation. The permeability to testosterone was decreased following DHA supplementation and the lipid profile was improved. Differences in the expression of the tight junction (TJ) proteins claudin-1, claudin-4, occludin, and TJ protein-1 were observed, principally a significant increase in claudin-1 expression, which was furthermore confirmed by Western blot analyses. In conclusion, these results confirm that the DHA supplementation of cell culture media modulates different aspects of skin barrier function in vitro and reflects the importance of n-3 PUFAs regarding the lipid metabolism in keratinocytes.

## 1. Introduction

The skin forms the interface between the body and the external environment. Its main function is to protect the organism against extraneous aggressions by forming a vital and waterproof barrier, thus preventing the onset of an immune response [1]. In addition, the skin prevents dehydration by limiting the diffusion of water outside of the organism. The barrier function is mainly provided by the outermost layer of the skin, namely the epidermis, and more precisely by the *stratum corneum* (SC) [2]. Keratinocytes differentiate in order to reach a terminal state in the SC, where they are called corneocytes. During the final stages of epidermal differentiation, keratinocytes undergo significant morphological changes, including the replacement of the plasma membrane by a rigid and insoluble protein shell, the cornified envelope [3]. The corneocytes are embedded in a lipid matrix, which ensures the maintenance of an optimal paracellular skin barrier function. The three main lipids that dominate the SC lipid matrix are cholesterol, free fatty acids, and ceramides, which adopt a highly ordered structure. Thus, they are arranged perpendicularly to the surface of the corneocytes, parallel to each other, on the extracellular side of the cornified envelope [4]. Additionally, tight junctions (TJs) are another major determinant of paracellular permeability, which is mainly provided by their integral membrane proteins, the claudins (Cldns) [5]. TJs control the flow of molecules through the intercellular space between the cells of the epidermis, so that compounds must enter the cells by diffusion or active transport in order to pass through the tissue [6]. Regarding Cldns, they constitute both paracellular barriers and pores in the skin, as they allow cell–cell contacts between neighboring keratinocytes [6,7]. They polymerize in a linear manner and form TJ strands with channel functions, which are robust and flexible, and which influence biological activities. To this day, 24 different isotypes of Cldns have been identified, but claudin-1 (Cldn-1) and claudin-4 (Cldn-4) are the most studied ones in healthy and diseased skin [8,9]. Moreover, occludins (Oclns), which are NADH oxidases that influence critical aspects of keratinocyte metabolism, are also important in TJ assembly and stability [10]. In counterpart, the tight junction protein-1 (ZO-1) is a cytoplasmic protein of the MAGUK (membrane-associated guanylate kinase homologs) family. This last protein contains a binding domain to TJ proteins, adherent junctions, and actin cytoskeleton [11]. This latter binding is involved in the role of ZO-1 as a scaffolding protein [12].

Polyunsaturated fatty acids (PUFAs) are essential fatty acids for the skin barrier, since alpha-linolenic acid (ALA) and linoleic acid (LA), the two precursors of n-3 PUFAs and n-6 PUFAs respectively, were found to be crucial for optimal skin homeostasis, including complete SC formation [13]. ALA and LA enter parallel pathways that utilize the same elongation and desaturation enzymes, converting them into eicosapentaenoic acid (EPA) and arachidonic acid (AA), respectively [14]. Afterwards, EPA can be transformed into DHA [15,16]. Each of these fatty acids can further be transformed into bioactive lipid derivatives that also influence skin equilibrium [16]. As early as 1931, Wesson and Burr reported that n-3 and n-6 PUFA deficiency in rats increased the metabolic rate of the body and decreased the impermeability of the skin [14,17]. Over the last decade, a variety of studies have proven the efficiency of a PUFA-rich diet in optimizing the skin barrier function [18,19,20]. In fact, the physical structure of the barrier function is dependent on LA, since it was reported that the SC contains large amounts of sphingolipids rich in linoleate content [21]. Additionally, bioactive lipids derived from both n-3 and n-6 PUFAs regulate several biological pathways in the skin and might be involved in the formation of the SC, even though the exact underlying mechanisms are still not understood [18,22].

In vitro tissue-engineered skin models are now well recognized as efficient tools for studying the pharmacology of therapeutic molecules, as well as the in vitro metabolization of various drugs [23,24,25]. Skin models such as these are particularly useful in dermatological industries, as they pave the way for more personalized and standardized tests, which can eventually lead to better results in terms of developing treatments for patients [26]. Over the last 20 years, our research team has developed a tissue-engineered method for the production of skin substitutes, using primary fibroblasts and keratinocytes [27,28]. We previously found that the use of ALA-supplemented culture media decreased the permeability to testosterone of our tissue-engineered skin model, thus promoting a more complete skin barrier function [18]. Interestingly, in this latter study supplementation with ALA did not translate into higher DHA levels although DHA is the predominant n-3 PUFA found in the phospholipids of normal human skin (NHS) [29]. Since testosterone permeability was still found to be higher in ALA-supplemented skin substitutes compared with NHS, we hypothesized that the use of long-chain n-3 PUFAs could have a bigger impact on the barrier function of skin substitutes. The tissue-engineered skin model produced with the self-assembly method has the advantage of containing as few exogenous materials as possible, meaning that the extracellular matrix of the dermis is produced by the stimulation of human fibroblasts in order to obtain manipulable dermal sheets. Furthermore, tape stripping of the SC to mimic desquamation was not performed in order to avoid adding any biases during the production of the skin substitutes. Thus, the self-assembly method used for this study is the most efficient for obtaining reproducible results that can be compared with our previous studies [26]. As such, we were able to compare the testosterone permeability and lipid profiles of our skin substitutes when produced with different supplements. Therefore, the aim of the present study was to evaluate the capacity of docosahexaenoic acid (DHA) to improve the impermeability of our 3D skin model, as well as its influence on the expression of proteins involved in TJs, in particular the Cldns. We showed in this study that the use of DHA-supplemented skin substitutes produces an improved lipid profile as compared with ALA-supplemented skin substitutes, in addition to improving testosterone permeability to similar fluxes. Moreover, to our knowledge, we have documented for the first time the modulation of the expression of TJ proteins by DHA in a tissue-engineered skin model. Our results concerning lipid profile and TJ proteins set this study apart and highlight some new advances in our understanding of the role of PUFAs and the mechanisms regulating skin barrier function, since few studies have shown the effect of PUFAs on TJ protein expression in the skin until now. In recent years, it has become evident that TJs are an important parameter for assessing skin barrier function, more precisely the proteins associated with TJs discussed in this study [30].

## 2. Results

### 2.1. Impact of DHA on the Morphology of the Skin Substitutes

**Figure 1:**
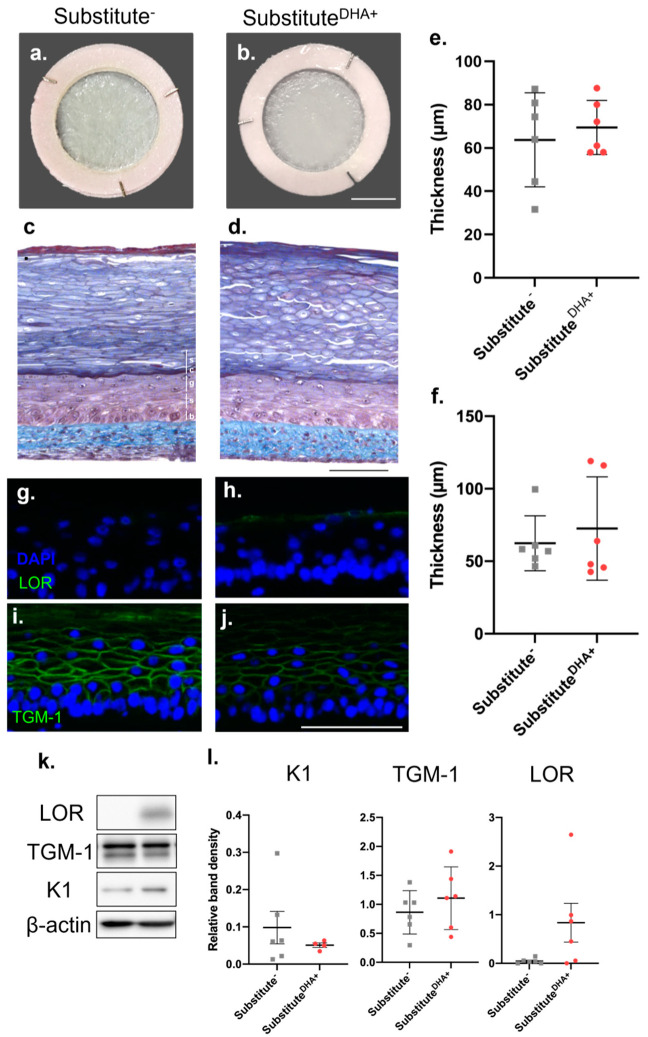
Influence of DHA supplementation on the skin substitutes’ morphology. (**a**,**b**) Macroscopic aspect and (**c**,**d**) histological cross-section following Masson’s trichrome staining of the skin substitutes. (b: basal layer, s: spinous layer, g: granular layer and sc: compact layers of the stratum corneum.) Scale bars: (**a**,**b**) 1 cm, (**c**,**d**,**g**–**j**) 100 μm. (**e**) Epidermal and (**f**) dermal thickness quantified from Masson’s trichrome-stained sections using ImageJ software. (**g**,**h**) Immunofluorescence staining of Lor (green) and (**i**,**j**) TGM-1 (green) with nuclei stained in blue with DAPI. (**k**) Western blot analysis of differentiation markers in the epidermis of skin substitutes followed by (**l**) quantification of signals for relative band densities using ImageJ Software. *n* = 6 (3 donors, 2 skin substitutes per donor, 3 measurements per skin substitute). The *p*-values were derived from Student’s t-tests and considered statistically significant when *p* < 0.05. Abbreviations: Lor, loricrin; TGM-1, transglutaminase-1; K1, keratin 1.

The macroscopic aspect of both unsupplemented skin substitutes (substitute^-^) and skin substitutes produced with the addition of DHA (substitute^DHA+^) presented a smooth and uniform surface after 21 days of culture at the air–liquid interface (Figure 1a,b). Histological analyses of substitute^-^ and substitute^DHA+^ showed that both conditions reproduce a similar cutaneous morphology (Figure 1c,d). The presence of a fully developed SC in both substitute^-^ and substitute^DHA+^ confirms that the keratinocytes differentiated correctly. The living epidermal thickness of substitute^DHA+^, which excludes the SC, and the dermal thickness were similar to the respective thicknesses of substitute^-^ (Figure 1e,f). The addition of DHA to the culture media modified the expression of differentiation proteins in the skin substitutes, as seen in Figure 1g–j. However, Western blot analyses did not show significant changes in the expression of differentiation markers between substitutes^DHA+^ and substitutes^−^ (Figure 1k,l).

### 2.2. Impact of DHA on the Permeability of the Skin Substitutes

**Figure 2:**
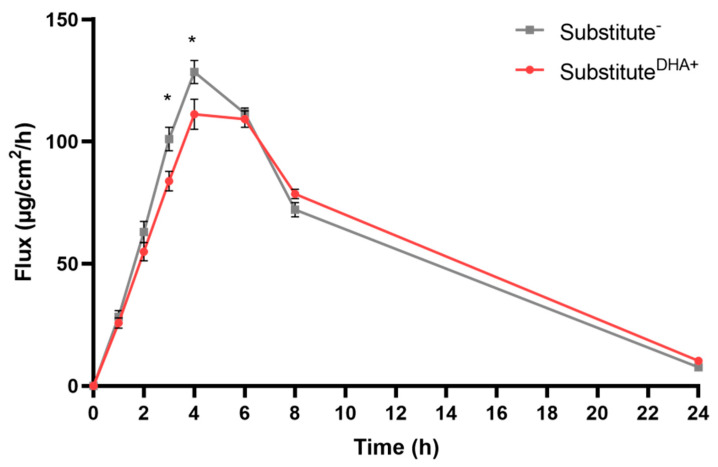
Testosterone permeability through skin substitutes with or without DHA supplementation of culture media. The Franz cell diffusion system was used to perform percutaneous absorption studies. The testosterone dosing solution was freshly prepared in ethanol/water (1:1), yielding a concentration of 4.0 mg/mL. Quantification was done with a Waters Acquity UPLC. Values are mean +/− standard error of the mean (SEM). *n* = 6 (3 donors, 2 skin substitutes per donor); *p*-values were derived from Student’s *t*-tests. * *p* < 0.05.

Testosterone permeability assays were used to study the percutaneous absorption of lipophilic molecules through the skin substitutes produced with or without DHA supplementation (Figure 2). Our results showed an enhanced barrier function in substitutes^DHA+^ compared with substitutes^−^. Both substitutes^DHA+^ and substitutes^−^ saw their mean fluxes of testosterone rapidly increase for the first four hours after testosterone application, where they reached their peak values. The substitutes^−^ showed increased permeability to testosterone compared with substitutes^DHA+^, with a significantly higher flux at the third and fourth hours after testosterone was applied to the epidermis (1.2-fold for each). Then, at the sixth hour, the fluxes started decreasing for both conditions up to 24 h (Supplementary Table S2). Thus, the barrier function of skin substitutes was improved with the addition of DHA to the cell culture media.

### 2.3. Impact of DHA on the Fatty Acid Phospholipid Profile of the Skin Substitutes

**Figure 3:**
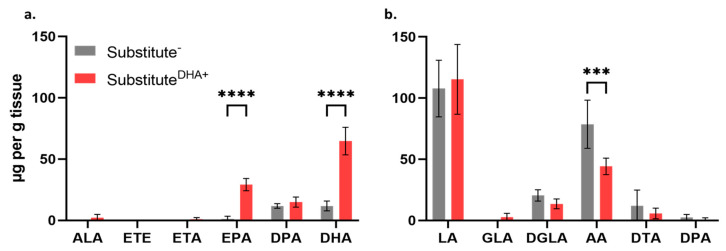
Incorporation of PUFAs in the phospholipid fraction of the epidermis of skin substitutes. Influence of the supplementation of the culture media with DHA on the levels of n-3 and n-6 PUFAs in the epidermal phospholipids of substitutes^−^ and substitutes^DHA+^ (**a**) n-3 PUFA levels; (**b**) n-6 PUFA levels. PUFAs were quantified by gas chromatography and results are presented as μg per g of tissue. One-way ANOVA followed by Sidak’s multiple comparison test. (*** *p*-value < 0.001; **** *p*-value < 0.0001). Abbreviations: AA, arachidonic acid; ALA, α-linolenic acid; DGLA, dihomo-γ-linolenic acid; DHA, docosahexaenoic acid; DPA, docosapentaenoic acid; DTA, docosatetraenoic acid; EPA, eicosapentaenoic acid; ETA, eicosatetraenoic acid; ETE, eicosatrienoic acid: GLA: γ-linolenic acid; LA, linoleic acid; PUFAs, polyunsaturated fatty acids.

The incorporation of DHA into the phospholipid fraction of the epidermis of substitute^-^ and substitute^DHA+^ was evaluated using the gas chromatography with flame ionization detector technique (GC-FID)(Supplementary Table S1). Regarding the n-3 PUFA metabolism, the addition of DHA to the culture medium resulted in significantly increased levels of EPA (21.8-fold) and DHA (5.5-fold) in the substitutes^DHA+^ compared with the controls (Figure 3a). Conversely, the exogenously added DHA induced a 1.8-fold decrease in the epidermal levels of AA in substitute^DHA+^ as compared with substitute^−^ (Figure 3b).

### 2.4. Impact of DHA on Epidermal Expression of TJ Proteins

**Figure 4:**
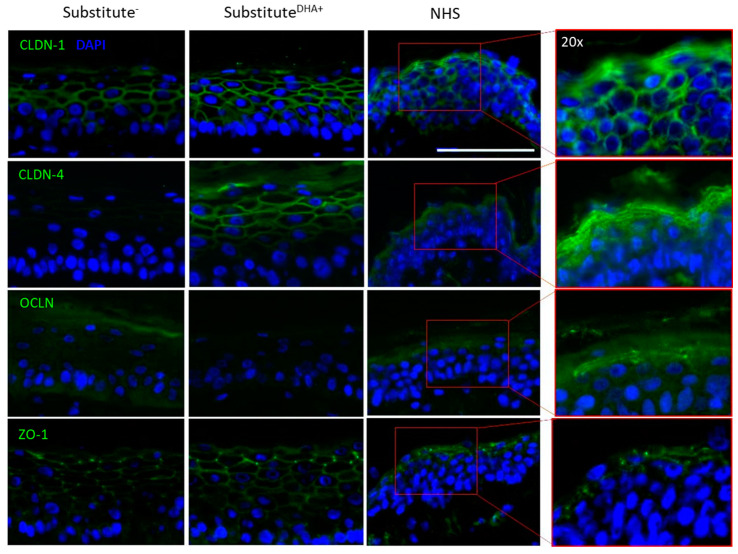
Expression of TJ proteins in the epidermis of skin substitutes. Immunofluorescence analyses were used to observe TJ protein expression patterns in the epidermis of skin substitutes. Cldn-1, -4, Ocln, and ZO-1 were stained in green and the nuclei were stained in blue with DAPI. Scale bars: 100 μm. *n* = 3 (3 donors, 2 skin substitutes per donor). Abbreviations: Cldn-1, claudin-1; Cldn-4, claudin-4; NHS, normal human skin; Ocln, occludin; ZO-1, tight junction protein-1.

To determine whether the effects of DHA on barrier function extend to tight junctions, the expression of TJ proteins in the skin substitutes was evaluated by indirect immunofluorescence and Western blot analyses. Indirect immunofluorescence analyses showed that Cldn-1, Cldn-4, Ocln, and TJ protein-1 (ZO-1) expression was slightly modulated following DHA supplementation, as seen in Figure 4. DHA supplementation tended to increase Cldn-1 expression. Moreover, immunofluorescence results showed a tendency of Cldn-4 and ZO-1 to be slightly increased in substitutes^DHA+^ compared with substitutes^−^. However, Ocln seemed to be reduced in substitutes^DHA+^.

**Figure 5:**
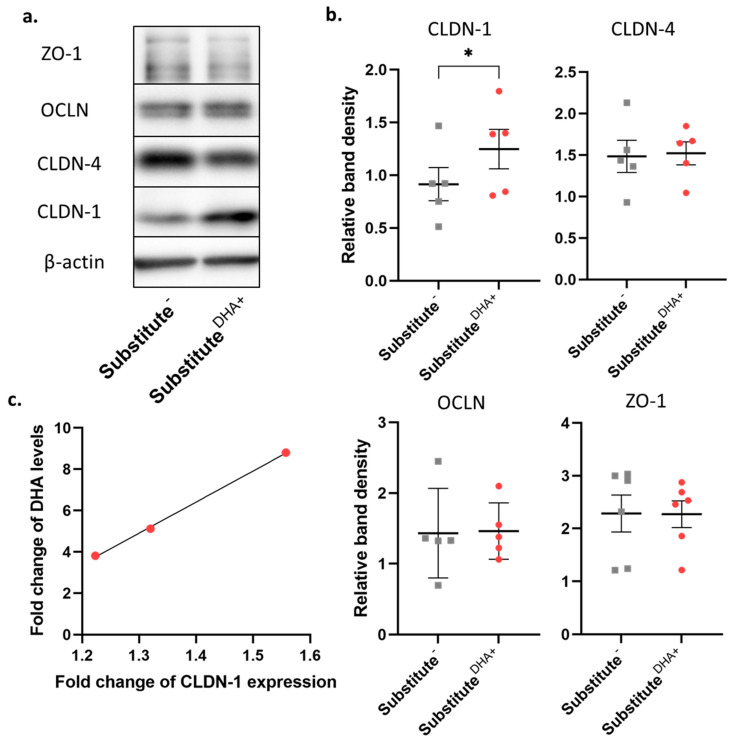
Levels of TJ proteins found in the epidermis of skin substitutes. (**a**) TJ molecules Cldn-1, Cldn-4, Ocln, and ZO-1 were analyzed by Western blotting. (**b**) Signals for relative band densities were quantified using ImageJ Software. *n* = 5, *p*-values were derived from Student’s *t*-tests and considered statistically significant when * *p*-value < 0.05. (**c**) Correlation between fold change of DHA levels and fold change of Cldn-1 in skin substitutes. Abbreviations: Cldn-1, claudin-1; Cldn-4, claudin-4; NHS, normal human skin; Ocln, occludin; ZO-1, tight junction protein-1.

In order to confirm these latter observations, Western blot analyses were performed (Figure 5a). There was considerable variation in measurements among the different skin donors. Only Cldn-1 expression was found to be significantly increased after DHA supplementation compared with unsupplemented skin substitutes. Significant changes were not detected in the expression of any of the other TJ proteins studied (Figure 5b). In addition, a correlation was identified between fold changes of DHA and the fold changes of Cldn-1 expression in the skin substitutes produced from the cells of three different donors (Figure 5c). This correlation indicated that skin substitutes which incorporated higher levels of DHA compared with their baseline levels were associated with a more pronounced increase in Cldn-1 expression.

## 3. Discussion

Regulatory authorities must perform rigorous percutaneous absorption analyses in order to establish recommendations regarding exposure to molecules that come in contact with the skin [31]. Although the use of tissue-engineered skin models has overcome several difficulties regarding percutaneous absorption analyses, such as low skin bioavailability, reconstructed skin models still display a suboptimal barrier function compared with NHS [32]. In the present study, we have shown that the supplementation of the culture media with DHA improved the barrier function of the skin substitutes. Our study also demonstrated that the incorporation of DHA into the epidermal phospholipids of the skin substitutes mainly influenced the expression of claudin-1, which is involved in the regulation of the barrier function. The time spent at the air–liquid interface during our study is similar to the time needed for the epidermis to completely form in NHS, a duration that can also be found in other studies using different types of dermal compartments [33,34]. Some models can be found in the literature, however, where culture conditions were adjusted for a shorter duration at the air–liquid interface of 14 to 17 days [35,36,37,38].

N-6 PUFAs, and in particular LA, are well known for influencing the skin barrier function through their incorporation into ω-hydroxylated ceramides [39]. However, the impact of n-3 PUFAs on the barrier function is still not extensively documented. In a previous study by our team, we have shown that the supplementation of the culture media with ALA decreased the percutaneous absorption of testosterone through the skin substitutes [18]. In the same manner, we illustrated in the present study that the exogenous addition of DHA is also able to decrease the percutaneous absorption of testosterone through tissue-engineered skin substitutes. The concentration of DHA in the cell culture media of supplemented skin substitutes leads to a large increase in DHA levels in the epidermal phospholipids, in concordance with a previous study on AA- and EPA-treated cells by Mbarik et al. This latter study reported that PUFAs are first stocked into phospholipids, and the authors hypothesized that with high dose supplementation of PUFAs, the phospholipids became saturated and PUFAs were then stocked into triglycerides [40]. Therefore, in the present study, we used a concentration of PUFA that allowed the formation of viable and morphologically adequate substitutes. To our knowledge, this study is the first to report that DHA can influence the establishment and maintenance of in vitro skin barrier function. Our results are concomitant with findings by Xiao et al., showing the improvement of intestinal barrier function following DHA supplementation in porcines, mainly by suppressing necroptosis TNF-alpha signaling [41]. Another study has shown that interleukin-10-deficient mice treated with DHA presented attenuated characteristics of colitis, mainly due to an enhanced intestinal epithelial barrier function [42].

The study by Simard et al. revealed that supplementation of the culture media with ALA did not increase the levels of DHA significantly, suggesting that the conversion rate of ALA to DHA was slow, as seen in skin in vivo [43]. Here, we showed that DHA supplementation allows its incorporation into the membrane phospholipids, leading to a phospholipid fatty acid profile more representative of NHS, as compared with ALA supplementation. In the present study, increased levels of EPA were measured following DHA supplementation, showing that DHA was efficiently retro-converted into EPA in our skin model. The metabolization of DHA also decreased the amounts of AA measured in the skin substitutes, suggesting that competition for the conversion enzymes is present for long-chain fatty acids [44,45]. These results are in accordance with a paper from Kendall et al. in which they reported a modulation of the levels of DHA metabolites in the epidermis after DHA oral supplementation, indicating that supplementation with DHA in vivo does affect its conversion in bioactive derived metabolites [46]. Since LA is recognized as an essential component for the maintenance of the epidermal water barrier, it can be hypothesized that the promotion of n-3 PUFA metabolism via DHA maintains endogenous levels of LA in the skin substitutes, thus increasing impermeability [14]. In addition to modulating the lipid profile of reconstructed skin substitutes, in many studies, DHA has been shown to have the capacity to influence epidermal protein expression. Recently, Jia et al. demonstrated that DHA increases the expression of filaggrin and Lor in a human reconstructed skin model [15]. This latter study is in line with our results, which show that DHA increased the expression of Lor and reduced the expression of TGM-1 in the epidermis of reconstructed skin tissues. Consequently, we hypothesized that the epidermal expression of other proteins implicated in the barrier function could also be influenced by the presence of DHA in the cell culture media.

Besides their influence on the composition of the SC lipid matrix, TJs are also known to play an important role in the barrier function of the human epidermis [47,48]. Interestingly, Cldn-1 is particularly recognized as a TJ protein that influences skin permeability [49]. Indeed, Cldn-1 knockout mice die soon after birth due to an ineffective skin barrier and consequent dehydration [5]. Accordingly, the down-regulation of Cldn-1 has been associated with impaired skin barrier function, enhanced skin permeability, and skin pathologies, such as psoriasis and atopic dermatitis [50,51,52,53,54]. Consistent with our current findings, an increased expression of Cldn-1 was also linked to improved skin barrier function in other human reconstructed skin models [55,56]. Furthermore, the ability of n-3 PUFAs to increase Cldn-1 expression has also been reported in other human and mouse models [57]. Moreover, n-3 PUFAs are known to influence the expression of TJ proteins, since the expression of TJ protein 1 (ZO-1), Cldn-1, Cldn-5, and Cldn-8 was found to be increased in a mouse model after n-3 PUFA oral supplementation [58,59]. However, the lack of significant changes in the expression of Cldn-4, Ocln, and ZO-1 observed in our study indicates that these proteins are not involved in the increased impermeability provided by DHA supplementation. The results showed considerable variation in measurements among the different skin donors. Variabilities such as gender, age, and skin phototype were previously documented to affect the barrier function and could influence the expression of tight junction proteins [60,61]. These factors are known to have an effect on percutaneous absorption, thus it would be of interest to carry out a thorough comparison of their impact on tissue-engineered skin substitutes in future studies.

Of note, the integrity of TJs was not affected by the incorporation of n-3 PUFAs in the membrane phospholipids [57]. Specifically, DHA has been shown to increase Cldn-1 expression in a mouse model of ulcerative colitis, in the intestinal cells of LPS-challenged piglets, and in TNF-alpha-challenged porcine epithelial cells [41,62,63,64]. Cldn-1 is known to be expressed in all epidermal layers, but its expression increases from the basal layer to the granular layer, where its expression reaches its peak. However, only one layer of the granular layer expressing Cldn-1 is known to play an active role in the epidermal barrier function [65]. Moreover, as seen in Figure 5c, our results showed that fold changes in Cldn-1 expression are correlated with fold changes in DHA levels found in skin substitutes produced from the cells of three different donors, meaning that DHA leads to improved barrier function partly through the reestablishment of Cldn-1 expression. Several hypotheses have been raised to explain the influence of DHA on Cldn-1 expression. DHA is a PPAR gamma agonist, which can regulate the differentiation of human keratinocytes in culture [45]. In addition, it has been suggested that the PPAR gamma activator upregulates the barrier function of epithelial cells by increasing the expression of tight junction proteins in vitro [66,67]. It has also been proposed that n-3 PUFAs modify TJ protein expression by redistributing TJ proteins into membrane microdomains, thus changing the lipid membrane composition, and reducing the permeability of epithelial cells [68]. In vitro studies have also demonstrated that Cldn-1- and Ocln-deficient keratinocytes showed decreased transepithelial electrical resistance as compared with controls. In addition, they showed that Cldn-1 knock-down cells affected Ocln localization in the cell membrane [69]. In addition, the expression of Cldn-4 was found to be increased in several pathological tissues with impaired barrier function, while decreased in other pathological tissues such as lobular carcinoma, suggesting that the expression of Cldn-4 is versatile and tissue dependent [70,71,72,73]. Regarding the localization of Cldn-4 and ZO-1, their expression also increases towards the outermost layers of the epidermis, but only starts in the spinous layer. Moreover, Ocln is specifically expressed in the granular layer, which corresponds to the results obtained in our study [74].

## 4. Materials and Methods

### 4.1. Cell Culture

This study was approved by the institutional review board of Université Laval. The volunteers signed the consent form in accordance with the Helsinki declaration and the guidelines of the Research Ethics Committee of the CHU de Québec–Université Laval. The breast reduction biopsies were obtained from three Caucasian women aged between 18 and 49 years old (cell populations: ♀18, ♀46, ♀49) and the fibroblasts and keratinocytes were extracted using the method based on thermolysin and trypsin [75]. Once efficiently extracted, they were stored in liquid nitrogen until their seeding.

### 4.2. Production of Tissue-Engineered Skin Substitutes

Skin substitutes were produced following the self-assembly approach described in previous studies (Supplementary Figure S1) [18,45,76]. Human primary fibroblasts (passage 6) were seeded at 1.25 × 10^4^ cells/cm^2^ in Dulbecco–Vogt modified Eagle’s medium (DME) supplemented with 10% fetal calf serum (Seradigm, Radnor, PA, USA) with 50 g/mL of ascorbic acid (Sigma, Oakville, ON, Canada), 0.06 mg/mL penicillin G (Sigma, Oakville, ON, Canada) and 25 µg/mL gentamicin (Schering, Pointe-Claire, QC, Canada) in 6 well-plates. After 25 days, fibroblast sheets were superimposed in pairs and cultured for three days to produce dermal equivalents. Human primary keratinocytes were then seeded at 1.2 × 10^6^ cells per dermal equivalent. DME mixed with Ham’s F12 medium (3:1) (Gibco, Life technologies, New York, NY, USA) and supplemented with 5% FetalClone II serum (Hyclone, Logan, UT, USA), 5 μg/mL insulin (Sigma, Oakville, ON, Canada), 0.4 μg/mL hydrocortisone (Galenova, St-Hyacinthe, QC, Canada), 0.4 μg/mL isoproterenol (Sigma, Oakville, ON, Canada), 10 ng/mL human epidermal growth factor (EGF) (Ango Inc, San Ramon, CA, USA), 60 μg/mL penicillin and 25 μg/mL gentamicin was then used as culture media. Cell culture media were changed thrice a week all throughout cell culture and the skin substitutes were kept at 37 °C in an 8% CO_2_ atmosphere.

### 4.3. Fatty Acid Supplementation of Cell Culture Media

Supplementation with DHA (Sigma, Oakville, ON, Canada) was carried out starting from fibroblast seeding in 6-well plates until biopsies were obtained. A solution of 100 mg DHA in 1 mL ethanol (EtOH) 99% was evaporated to determine volumes to produce a total concentration in cell culture media of 10 μM. This concentration was determined following the results of previous dose-response studies on the effect of cell culture media PUFA supplementation on skin morphology and cell proliferation [18,40]. Once the EtOH was successfully evaporated, serum was added to dilute the DHA. Unsupplemented substitutes (substitutes^−^) were also produced as controls to determine if the barrier function of the skin substitutes was truly affected by the presence of n-3 fatty acids in cell culture media.

### 4.4. Histological Analysis

Masson’s Trichrome staining was used to prepare sections for the histological analysis of two skin substitutes for each of the three donors (*n* = 6). Firstly, skin substitutes were fixed in a HistoChoice solution (Sigma, Oakville, ON, Canada) before being embedded in paraffin. They were then cut in 5 μm thick sections and stained with Masson’s Trichrome staining. The thickness of the epidermis was measured with Image J software (National Institutes of Health (NIH), Bethesda, USA, http://imagej.nih.gov/ij (accessed on 29 September 2021)) by collecting ten measurements on three different sections of each stained biopsy (*n* = 18 for each condition).

### 4.5. Percutaneous Absorption

The testosterone permeability of in vitro skin substitutes was determined using the standard Franz diffusion cell technique, and was carried out according to the Organization for Economic Cooperation Development (OECD) guidelines [31,77,78]. Once skin substitutes were placed between the donor and receptor compartments of the diffusion cells (5 mL volume, 0.63 cm^2^ surface area; Crown Glass, Somerville, NJ, USA), the receptor compartments were filled with a 0.1 M phosphate buffered saline (PBS) solution at pH 7.4. A 4 mg/mL testosterone solution in EtOH/water (1:1, *v*/*v*) was prepared and 100 μL were added per donor compartment of the Franz diffusion cells, marking the initial time point (0). Parafilm was placed on the donor compartments to ensure occlusion and the temperature of the receptor compartments was set at 37 °C in order to produce a temperature of approximately 32 °C for the reconstructed skin substitutes. Samples were collected from the receptor compartments with a 5 mL syringe lengthened with a catheter (3 ½ Tom Cat Length 4 ½) at various time points (1 h, 2 h, 3 h, 4 h, 6 h, 8 h and 24 h), and filtered at 0.22 μm. Samples were finally kept at −20 °C until their analysis.

The quantification of testosterone in the samples was performed using a Waters Acquity UPLC system with a Waters photodiode array (PDA) detector and a thermostatted autoinjector (Acquity UPLC H-Class System, Waters, Mississauga, ON, Canada). An in-house-developed UPLC-UV method was used at 248 nm to successfully separate and detect the testosterone present in each sample. The separation was carried out using a BEH C18, Waters column (50 mm × 2.1 mm, 5 μm, Mississauga, ON, Canada) kept at 50 °C. The mobile phase was a concentration gradient of acetonitrile in water with 0.1% trifluoroacetic acid (TFA) eluted at a flow rate of 0.6 mL/min. Testosterone peaks eluted at 0.91 min under these conditions. Data were collected and peak integration was performed using Empower 2 software (Waters, Mississauga, ON, Canada).

### 4.6. Gas Chromatography

Gas chromatography was used to analyze the lipid profiles of the skin substitutes. This analysis was performed to quantify various lipids found in the phospholipid fraction of the epidermis. First, both the epidermis and dermis were separated using forceps and scalpels. The extraction of epidermal lipids following a modified Folch method required a chloroform/methanol mixture (2/1 *v*/*v*) [79]. Phospholipids were separated by thin layer chromatography with isopropyl ether/acetic acid (96/4 *v*/*v*) until the middle of the plate was reached. The fatty acids of isolated phospholipids were then methylated.

Thin layer chromatography was followed by gas chromatography using a HP5890 gas chromatograph (Hewlett-Packard, Toronto, ON, Canada) composed of an HP-88 capillary column (100 mm × 0.25 mm internal diameter × 0.20 μm film thickness; Agilent Technologies, Santa Clara, CA, USA) coupled with a flame ionization detector. Helium was used as carrier gas (split ratio: 1:80). The gas chromatography provided fatty acid profiles as described elsewhere [18,77].

### 4.7. Western Blot Analysis

The epidermis was separated from the dermis using a scalpel and forceps before being stored at −80 °C. Samples were crushed with a Cryomill MM400 (Retsch, Newtown, PA, USA) before proceeding to protein extraction with 500 μL of RIPA buffer containing the protease inhibitor cocktail cOmplete (Roche, Mannheim, Germany). Once the buffer was added to the samples, they were incubated on ice for 20 min and centrifuged at 4 °C for another 20 min at 12,000× *g*. The quantification of these extracts was performed with a Pierce BCA Protein Assay Kit (Thermo Fisher Scientific, Rockford, IL, USA).

Western blot analyses were performed to compare the expression of Cldn-1 (1:2000, ab15098, Abcam, Cambridge, MA, USA), Cldn-4 (1:1000, 329403, Thermo Fisher Scientific, Eugene, OR, USA), ZO-1 (1:50, 40-2200, Thermo Fisher Scientific, Eugene, OR, USA), Ocln (1:1000, ab216327, Abcam, Cambridge, MA, USA) and actin-β (1:30,000, Sigma, Oakville, ON, Canada) in supplemented and unsupplemented substitutes. To do so, total proteins (20 μg) were loaded onto 10% reducing SDS-PAGE gels. Once the migration was over, gels were transferred onto Immun-Blot PVDF membranes (Bio-Rad Laboratories, Mississauga, ON, Canada) at 25 Volts and 4 °C overnight. Membranes were blocked for 1 h in Tris-buffered saline 0.1% Tween-10 and 5% nonfat milk and incubated for 1 h with the primary antibodies and another hour with the secondary antibodies; either anti-mouse HRP (1:60,000, 115-035-003, Jackson Immuno Research Laboratories Inc., West Grove, PA, USA) or anti-rabbit HRP (1:60,000, 111-035-003, Jackson Immuno Research Laboratories Inc., West Grove, PA, USA) antibodies were used depending on the source of the primary antibodies.

### 4.8. Immunofluorescence Staining

The indirect immunofluorescence labeling of Cldn-1 (1:200, ab15098, Abcam, Cambridge, MA, USA), Cldn-4 (1:200, 329403, Thermo Fisher Scientific, Eugene, OR, USA), ZO-1 (1:50, 40-2200, Thermo Fisher Scientific, Eugene, OR, USA), Ocln (1:100, ab216327, Abcam, Cambridge, MA, USA), Lor (1:1600, 905104, BioLegend, San Diego, CA, USA), and TGM-1 (1:300, 12912-3-AP, Proteintech, Rosemont, IL, USA) was performed on previously embedded skin substitutes (Tissue-Tek OCT Compound (optimum cutting temperature); Sakura Finetek, Torrance, CA, USA). At this point, 5 μm thick sections of samples were cut using a Leica Cryostat before being placed on microscope slides. Acetone was used to fix tissues for 10 min at −20 °C. Once fixed, tissues were incubated in 1% BSA-PBS with the primary antibodies, then the secondary antibodies, which were either Alexa488 anti-rabbit (1:1600, A21206, Thermo Fisher Scientific, Eugene, OR, USA) or Alexa488 anti-mouse (1:1400, A11001, Thermo Fisher Scientific, Eugene, OR, USA), in a dark humidified chamber for 45 min and 30 min respectively. A mounting medium containing DAPI (Fluoromount-G; SouthernBiotech, Birmingham, AL, USA), which stains the cell nucleus, was used to mount the slides. Stained slides were imaged using a Zeiss microscope equipped with an AxioCam HR Rev3 camera (Carl Zeiss Meditec AG, Oberkochen, Germany).

### 4.9. Statistical Analysis

Data were expressed as means ± standard deviation for parametric variables, except when stated otherwise. Fatty acid lipid profiles were analyzed statistically using analyses of variance (ANOVAs) with Sidak’s post-hoc tests. Only values of *p* < 0.05 were considered significant. All statistics were calculated with the Prism version 9 software (Graphpad Software, La Jolla, CA, USA).

## 5. Conclusions

Collectively, our results show that DHA improves the barrier function of skin substitutes produced by tissue engineering, suggesting that long-chain n-3 PUFAs could be involved in maintaining optimal skin impermeability. In our study, the added DHA was properly incorporated into the phospholipid fraction of the epidermis and further metabolized into EPA, showing that our skin substitutes are metabolically active. Finally, our study also pointed out that DHA influences TJ proteins, resulting in an up-regulation of Cldn-1 that could potentially contribute to the improvement of the skin impermeability following DHA supplementation.

## Data Availability

The data presented in the study are directly available in the article.

## Supplementary Materials

The following are available online at https://www.mdpi.com/article/10.3390/ijms222313091/s1.
