# Supplementary material for: Docosahexaenoic Acid Modulates Paracellular Absorption of Testosterone and Claudin-1 Expression in a Tissue-Engineered Skin Model
Source: Int J Mol Sci. 2021 Dec 3;22(23):13091. doi: 10.3390/ijms222313091 (PMC8658185; doi:10.3390/ijms222313091)
Supplement: Supplementary file 1 [file ijms-22-13091-s001.zip › ijms-1429137-supplementary.pdf]

SUPPLEMENTARY MATERIAL

Table S1. Levels of epidermal phospholipid fatty acids following DHA supplementation.

	Epidermis		
	Mean ± SD (µg per g of tissue)		
**Fatty acids**	**Substitute⁻**	**Substitute^{DHA+}**	**p-value**
**Saturated FAs**			
14:0 (Myristic acid)	38,8 ± 11,0	33,7 ± 8,7	NS
16:0 (Palmitic acid)	292,5 ± 69,9	233,0 ± 62,2	NS
18:0 (Stearic acid)	236,2 ± 61,6	199,2 ± 45,9	NS
**n-3 PUFAs**			
18:3n-3(ALA)	0 ± 0	2,3 ± 2,6	NS
20:3n-3 (ETE)	0 ± 0	0 ± 0	NS
20:4n-3 (ETA)	0 ± 0	0,9 ± 1,4	NS
20:5n-3 (EPA)	1,3 ± 2,1	29,2 ± 5,0	<0,0001
22:5n-3 (DPA)	11,8 ± 1,9	14,9 ± 4,1	NS
22:6n-3 (DHA)	11,8 ± 4,0	64,7 ± 11,2	<0,0001
**n-6 PUFAs**			
18:2n-6 (LA)	107,8 ± 23,1	115,4 ± 28,5	NS
18:3n-6 (GLA)	0,0 ± 0,0	2,8 ± 3,0	NS
20:3n-6 (DGLA)	20,4 ± 4,6	13,6 ± 4,0	NS
20:4n-6 (AA)	78,6 ± 19,7	44,2 ± 6,7	0,0001
**n-7 MUFAs**			
16:1n-7 (Palmitoleic acid)	179,4 ± 56,3	152,4 ± 35,8	NS
18:1n-7 (Vaccenic acid)	317,6 ± 104,9	198,3 ± 72,7	0,0469
**n-9 MUFAs**			
18:1n-9 (Oleic acid)	603,5 ± 158,3	518,0 ± 85,9	NS
**Totals**			
Total PUFAs	291,7 ± 60,4	342,3 ± 54,3	NS
Total MUFAs	902,0 ± 571,4	716,2 ± 351,4	NS
Total FAs	2073,9 ± 511,2	1757,6 ± 347,9	<0,0001

Abbreviations : AA, arachidonic acid; ALA, α-linolenic acid; DHA, docosahexaenoic acid; EPA, eicosapentaenoic acid; FA, fatty acid; LA, linoleic acid; MUFA, monounsaturated fatty acid; PUFAs, polyunsaturated fatty acids; SFA, saturated fatty acid; SD, standard deviation.

Table S2. Flux of testosterone in skin substitutes following DHA supplementation.

Time (h)	Mean ± SEM (µg/cm²/h)		p-value
	Substitute⁻	Substitute^{DHA+}	
**1**	28,3 ± 2,6	25,8 ± 2,1	NS
**2**	63,0 ± 4,4	54,9 ± 3,8	NS
**3**	101,0 ± 4,8	83,8 ± 4,0	0,0101
**4**	128,5 ± 4,8	111,2 ± 6,2	0,0387
**6**	111,6 ± 2,2	109,2 ± 3,3	NS
**8**	72,2 ± 2,9	78,6 ± 1,9	NS
**24**	7,6 ± 0,6	10,3 ± 1,0	NS

All data are mean ± SEM. Statistical analyses were performed with two-way ANOVA followed by Sidak's post-hoc test. Abbreviation: SEM, standard error of the mean.

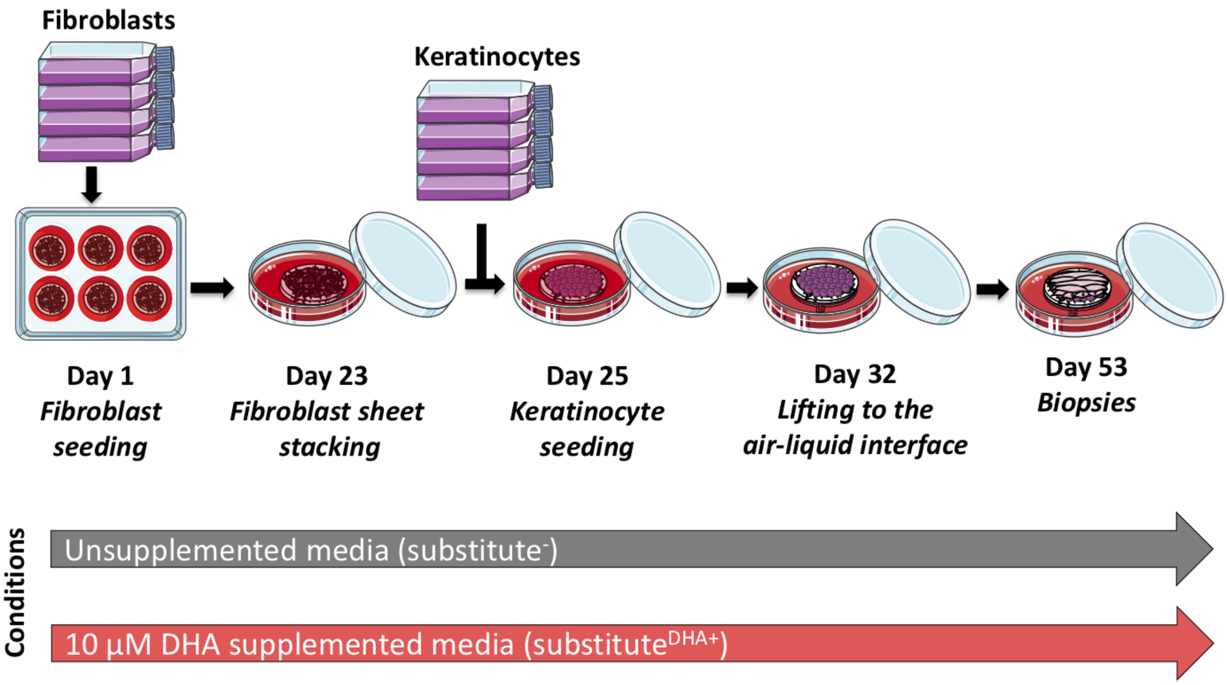

Figure S1. Schematic overview of the preparation of tissue-engineered skin substitutes according to the self-assembly method. A) Fibroblasts were cultured with ascorbic acid to form manipulatable sheets. **B**) Two fibroblast sheets were stacked to form the dermal layer. **C**) Keratinocytes were seeded on the dermal layer and skin substitutes were cultured for 1 week in submerged conditions and then **D**) for 21 days at the air-liquid interface. **E**) Biopsies were taken from the skin substitutes and were analyzed.

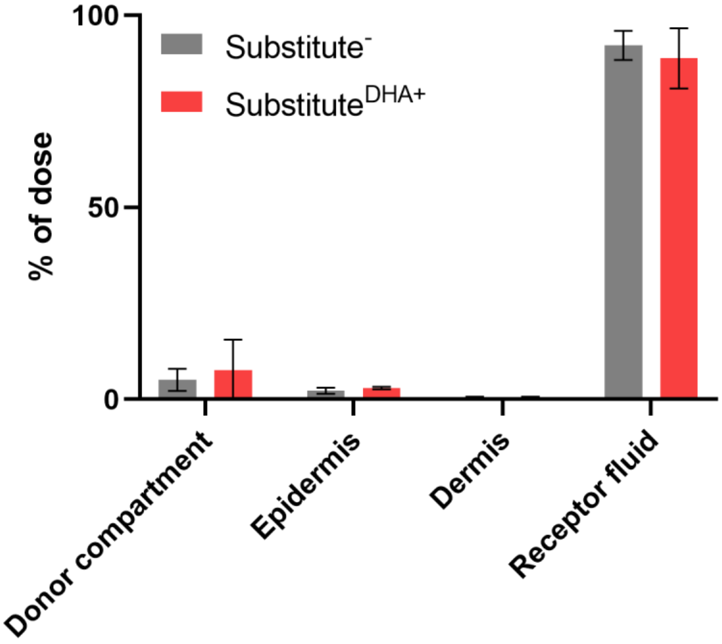

Figure S2. Percutaneous absorption of testosterone through skin substitutes. Testosterone was recovered in the different compartments after 24 h and is expressed as percentage of dose. Values are mean +/− standard error of the mean (SEM). n=15 (3 donors, 5 skin substitutes per donor), Two-way ANOVA followed by Tukey's *post-hoc* test.
